# Predicting object properties based on movement kinematics

**DOI:** 10.1186/s40708-023-00209-4

**Published:** 2023-11-04

**Authors:** Lena Kopnarski, Laura Lippert, Julian Rudisch, Claudia Voelcker-Rehage

**Affiliations:** 1 Department of Neuromotor Behavior and Exercise, Institute of Sport and Exercise Sciences, University of Münster, Wilhelm-Schickard-Str. 8, 48149 Münster, Germany; 2 Applied Functional Analysis, Chemnitz University of Technology, 09107 Chemnitz, Germany

**Keywords:** Kinematics, Arm movement, Object replacement, Prediction, Classification, Pattern recognition

## Abstract

In order to grasp and transport an object, grip and load forces must be scaled according to the object’s properties (such as weight). To select the appropriate grip and load forces, the object weight is estimated based on experience or, in the case of robots, usually by use of image recognition. We propose a new approach that makes a robot’s weight estimation less dependent on prior learning and, thereby, allows it to successfully grasp a wider variety of objects. This study evaluates whether it is feasible to predict an object’s weight class in a replacement task based on the time series of upper body angles of the active arm or on object velocity profiles. Furthermore, we wanted to investigate how prediction accuracy is affected by (i) the length of the time series and (ii) different cross-validation (CV) procedures. To this end, we recorded and analyzed the movement kinematics of 12 participants during a replacement task. The participants’ kinematics were recorded by an optical motion tracking system while transporting an object, 80 times in total from varying starting positions to a predefined end position on a table. The object’s weight was modified (made lighter and heavier) without changing the object’s visual appearance. Throughout the experiment, the object’s weight (light/heavy) was randomly changed without the participant’s knowledge. To predict the object’s weight class, we used a discrete cosine transform to smooth and compress the time series and a support vector machine for supervised learning from the achieved discrete cosine transform parameters. Results showed good prediction accuracy (up to $95\%$, depending on the CV procedure and the length of the time series). Even at the beginning of a movement (after only 300 ms), we were able to predict the object weight reliably (within a classification rate of $88-94\%$).

## Introduction

Recent motor control research has suggested that the central nervous system uses so-called forward models to simulate the sensorimotor consequences of an action prior to completing it [1]. Through this simulation, the sensorimotor consequences of an action can be anticipated and, if necessary, error corrections can be implemented with minimal delay. For example, when lifting or moving an object, the initial grip and load forces are scaled according to the anticipated properties of the object, such as its weight [2]. The object’s properties are typically anticipated based on previous experience and knowledge. When we, for example, expect that the milk carton we are about to pick up from a shelf is full, we anticipate that it will be certain weight and scale our grasp and load force appropriately before we pick it up. If, contrary to our expectation, the milk carton is empty, our forward model would be flawed resulting in excessive lifting velocity and height. To counter this, we activate feedback-error correction mechanisms. Information about an object’s weight can also be extracted from the observed kinematics of another person lifting or moving the object [3]. That is, heavy objects affect the movement kinematics differently than light objects. When handing an object from one person to another, this information is important for the receiver’s creation of an accurate forward model and, thus, increases the likelihood the joint action task will be completed successfully. Therefore, in this study, we investigate how changing the weight of an object is reflected in the movement kinematics of an actor during a pick-up and replacement task.

The prediction of the physical properties of objects also plays a major role in robotics [4]. It is assumed that, in the future, the use of robotic devices will increase in many fields including construction, manufacturing, and everyday assistance and care robots [5]. Assistance/care robots, in particular, pose the additional challenge of human–robot interactions. As a result, interactions with unknown objects within unknown environments will become increasingly important [6]. To guarantee reliable grasping, it is essential that the robot will be able to apply a suitable grip force. If too little grip force is applied, the object will slip and may be dropped. However, too much grip force can damage an object or lead to awkward object handovers between humans and robots. To date, determining an object’s weight before a robot has had physical contact it has been a major challenge and several possible solutions have already been proposed including image recognition [7] and thermography [4]. Another approach that could be important, especially in human–robot handover actions, is to predict the object’s weight by analyzing the human kinematics while they manipulate the object. This method could be used to provide a suitable online weight estimation before the robot’s first contact with the object and would, therefore, have an advantage over image recognition by being independent of whether the object class is already known or not.

Different object properties affect the kinematics in reach–grasp–manipulation tasks in different ways. The grip force required for a pick-up and replacement task is primarily dependent on the object’s weight. When grasping a known object, it is not only the target grip force that increases as object weight increases, the temporal pattern of grip force initiation (grip force development rate) increases as well. As the grip force development rate increases with object weight, the target grip force is generated at a similar duration, regardless of whether the object being lifted is light or heavy. Consequently, the duration between the first contact with the object and the start of the lifting movement should be independent of object weight [8]. However, this only seems to apply when the weight of the object is known. If the object’s weight is unknown or different than expected, this affects the grip force development rate in the time between first contact and the lifting of the object [9]. More precisely, if the object is heavier than expected, then the duration between first contact and the lifting phase is longer, and if the object is lighter than expected, then it is shorter [3, 10]. Although grasp duration seems to be dependent of the object weight, it could be shown that humans probably mainly consider the lifting velocity in the prediction of the object weight [3]. Therefore, it is reasonable to assume that observing how a person moves while lifting an object might provide information about the object’s weight that could facilitate appropriate grip force scaling even if the object’s weight is unknown or it cannot be detected reliably based on size alone. Therefore, we hypothesize that the movement kinematics following the grasp phase of a replacement task might provide the necessary information.

In this study, we investigated whether information about the weight (light/heavy) of an object can be determined based on the movement kinematics of an actor in an object pick-up and replacement task. In the past, essential information (pattern recognition) has been extracted from complex multidimensional kinematic data using various multivariate statistical models and machine learning algorithms, such as cluster analysis [11]. Our literature search identified manifold pattern recognition use cases in, for example, the design process for intelligent wearable devices [12], clinical gait analysis [13], and non-clinical biomechanical research [14]. One of the main goals of machine learning is to search for distinct patterns in movement kinematics in order to identify or qualitatively classify separate movement phases or actors. For example, previous research has shown that machine learning can be used to predict different disorders in the sensorimotor control system including Parkinson’s disease [15], cerebral palsy [16], and stroke [17]. These applications are, therefore, useful because they can identify certain degenerative disorders at an early stage, classify different subtypes of diseases based on their behavioral impact and assess therapeutic success. Furthermore, research on healthy individuals has shown that machine learning can be used to classify person’s emotional state [18] and to identify the specific movement characteristics of various throwing disciplines [19].

Many studies have focused on classifying gait and full-body kinematics. However, upper extremity tasks including object replacements and handover tasks have been less frequently investigated. Classifying upper limb movements may be particularly useful in interactions between humans and robotic agents. Social gestures, for example, can be classified with high accuracy [20] and, as such, may be used as an explicit form of communication with robots. It may also be possible to use machine learning to detect convention in human movements [21] which would allow robotic agents to anticipate human actions and develop a response plan/action more quickly. In addition, pattern recognition and classification may also be useful during joint action tasks, such as a handover, as it facilitates the predictive modeling of key parameters necessary to successfully participate in such collaborative tasks [22]. The time profiles of kinematic data can be used to predict key properties in such scenarios. It has already been shown that the object weight class can be predicted with an accuracy of around 0.35 above the odds in a handover action by using the giver’s kinematics [23]. A standardized experimental setup of invariant object start position was used in the former study. Therefore, it is still an open question whether object weight prediction is also possible when the kinematic data originates from experimental setups with greater variance by randomizing the object’s start position.

In this paper, we present a new approach in which we use cosine transformation for reduction of multidimensional movement kinematics data from a pick-up and replacement task in order to classify the weight class of the transported object (light/heavy) from it by use of a support vector machine (SVM). Two different types of kinematic data were extracted from the dataset: (1) we used the time series of the angles between upper body segments (upper/lower arm, hand), and (2) the velocity profiles of the object during object transport. Furthermore, we investigated the prediction accuracy i) in relation to time series of different lengths (i.e., to detect whether early weight prediction is possible) and ii) in relation to different cross-validation (CV) procedures based on a) random training/testing subsets over all participants or b) training/testing subsets that are split by participants. Ultimately, the results of this study will help to establish when and how accurately artificial agents can anticipate the weight class of an object based on the observed movement kinematics of an individual, information which, we anticipate, will be an advantage that facilitates using robotic devices as receivers of objects in joint handover tasks.

## Methods

### Participants

Twelve healthy participants (3 female) aged $24.2 \pm 1.7$ years (range 22–28 years) participated in the experiment. All participants had normal or corrected-to-normal vision. Two male participants were left-handed and all other participants were right-handed. All participants were students at the University of Münster, Germany, in the faculty of Psychology & Sport Sciences.

### Materials

#### Motion tracking

**Fig. 1:**
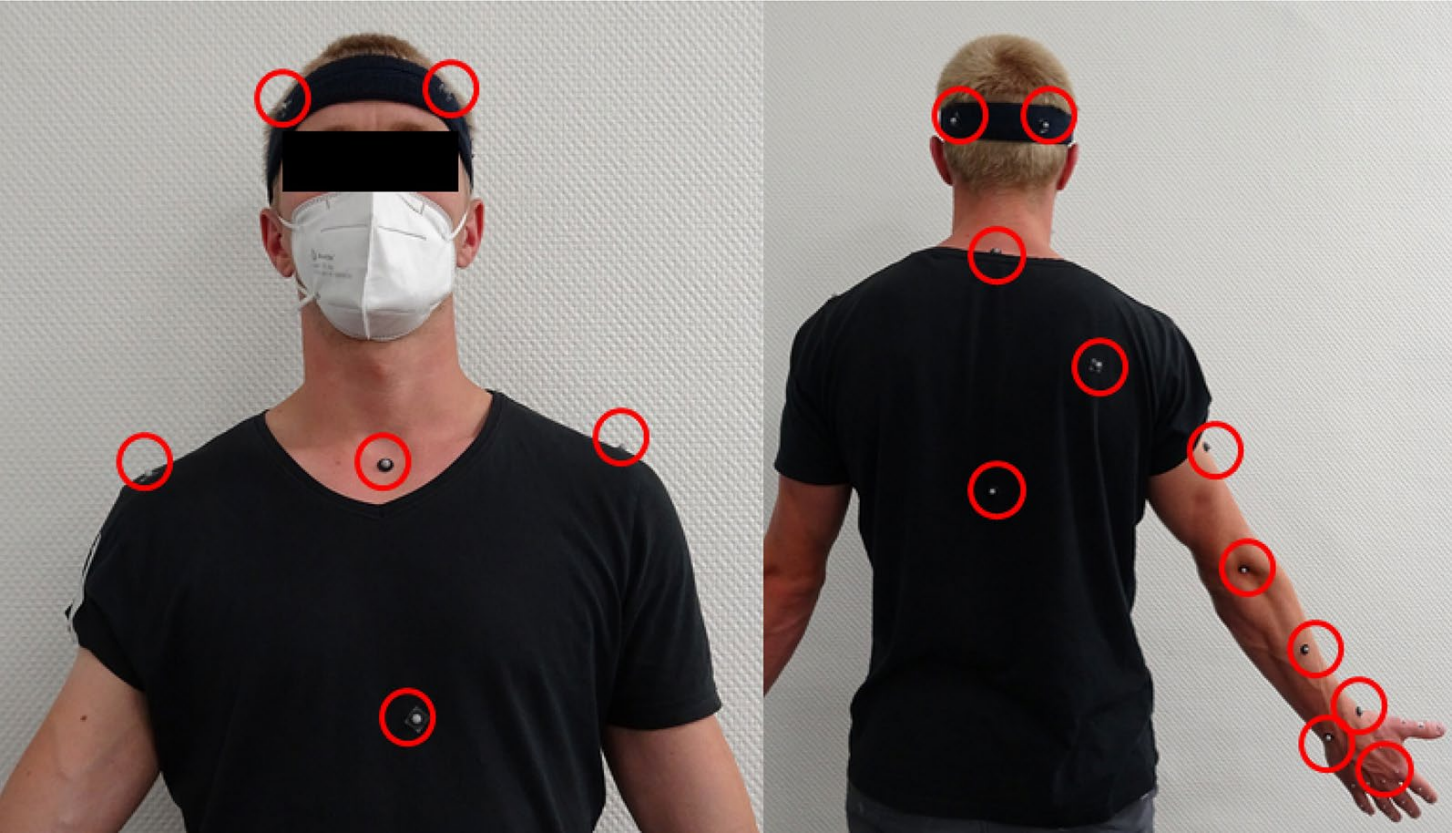
Marker setup. Positions of the 17 reflective markers (based on the Plug-in Gait model, Vicon, 2022)

A passive marker-based optical motion capture system (Vicon Motion Systems Ltd, Oxford, UK) with 10 cameras was used to record the participants’ motions and the motion of a test object at a sampling frequency of 100 Hz. Seventeen spherical reflective markers with a diameter of 6.4 mm were attached to the participant’s upper body (head, trunk, shoulders, right arm; see Fig. 1). The marker set was based on the Plug-in Gait model [24]. The following upper body angles were extracted (with Nexus [24]) for the right arm: between the upper body and upper arm (flexion/extension, abduction/adduction, internal/external rotation), the upper arm and forearm (flexion/extension), the forearm and hand (flexion/extension, ulnar/radial deviation), and the hand rotation along the longitudinal axis of the forearm (identified by the Plug-in Gait as wrist rotation).

#### Test object

**Fig. 2:**
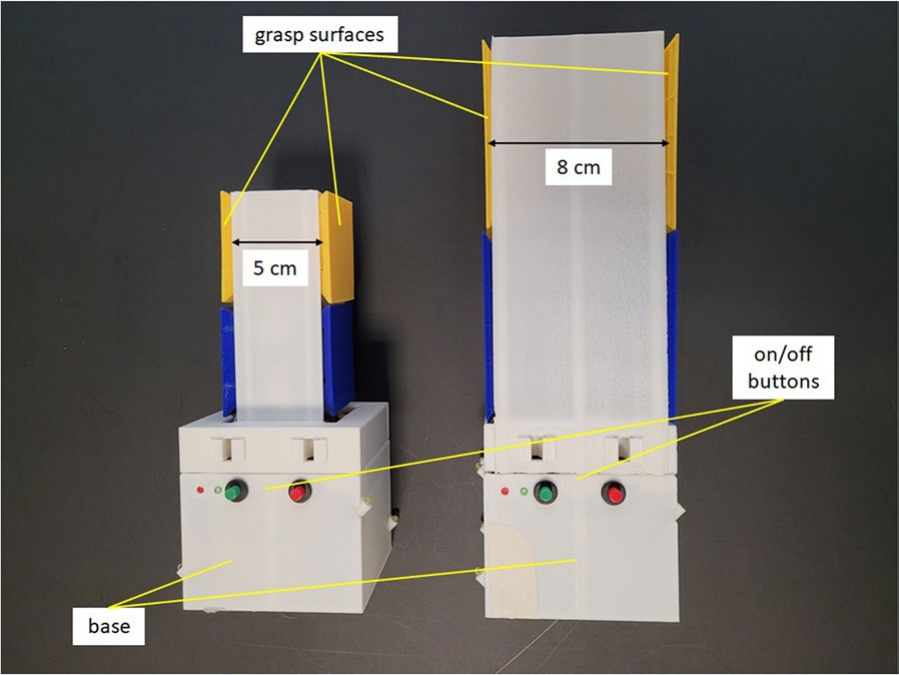
Test objects. Small (left) and large (right) object with a base of ${8}\,\hbox {cm} \times {8}\,\hbox {cm} \times {8}\,\hbox {cm}$ and grasping surfaces of ${5}\,\hbox {cm} \times {5}\,\hbox {cm} \times {5}\,\hbox {cm}$ or ${8}\,\hbox {cm} \times {8}\,\hbox {cm} \times {8}\,\hbox {cm}$, respectively. A weight of 502 g can be embedded in the base body

A self-constructed, 3D-printed test object was used to assess upper body movements during a replacement task. This test object included four force transducers for the measurement of grip forces (not used for this study) and five infrared LEDs (embedded in the base) which allowed the object’s motion to be tracked in the Vicon system. Two different size test objects were used. Both objects had an identical base (${8}\,\hbox {cm} \times {8}\,\hbox {cm} \times {8}\,\hbox {cm}$) housing the LEDs and in which a weight (502 g) could be attached inside the object without changing the exterior appearance. The grasping surfaces of the two objects differed in size and distance from each other (${5}\,\hbox {cm} \times {5}\,\hbox {cm} \times {5}\,\hbox {cm}$; ${8}\,\hbox {cm} \times {8}\,\hbox {cm} \times {8}\,\hbox {cm}$) and were arranged one on top of the other on top of the base (see Fig. 2). Only the upper (yellow) grasping surfaces were used for this study. The small object weighed 341 g in the light condition (without attached weight) and 843 g in the heavy condition (with attached weight). The large object weighed 372 g in the light condition (without attached weight) and 874 g in the heavy condition (with attached weight). In order to provide more variance in the kinematics (besides the starting position of the object), which is independent of the object weight class, half of the participants performed the pick-up and replacement task with the small object size, and the other half with the large object size.

#### Task and procedures

We designed a pick-up and replacement task, in which the participant grasped an object in a defined start position and placed it in a specific end position. Two objects, different in size and weight, were used to capture the upper body kinematics in relation to the object’s weight class. Markers were attached to the participants’ trunk and upper arm according to the Plug-in Gait marker set (see Fig. 1). Participants were seated at a table on a height-adjustable stool. The stool was adjusted so that the elbow was bent at $90^\circ$ when the forearm was placed on the table and the palm of the hand rested flat on the table. Participants were asked to take this position at the beginning and end of each trial. Before commencing the experimental trial, the setup was calibrated to the participant’s specific model by taking a static measurement in the rest position followed by a dynamic measurement that involved the participant moving all the joints in their arm.

**Fig. 3:**
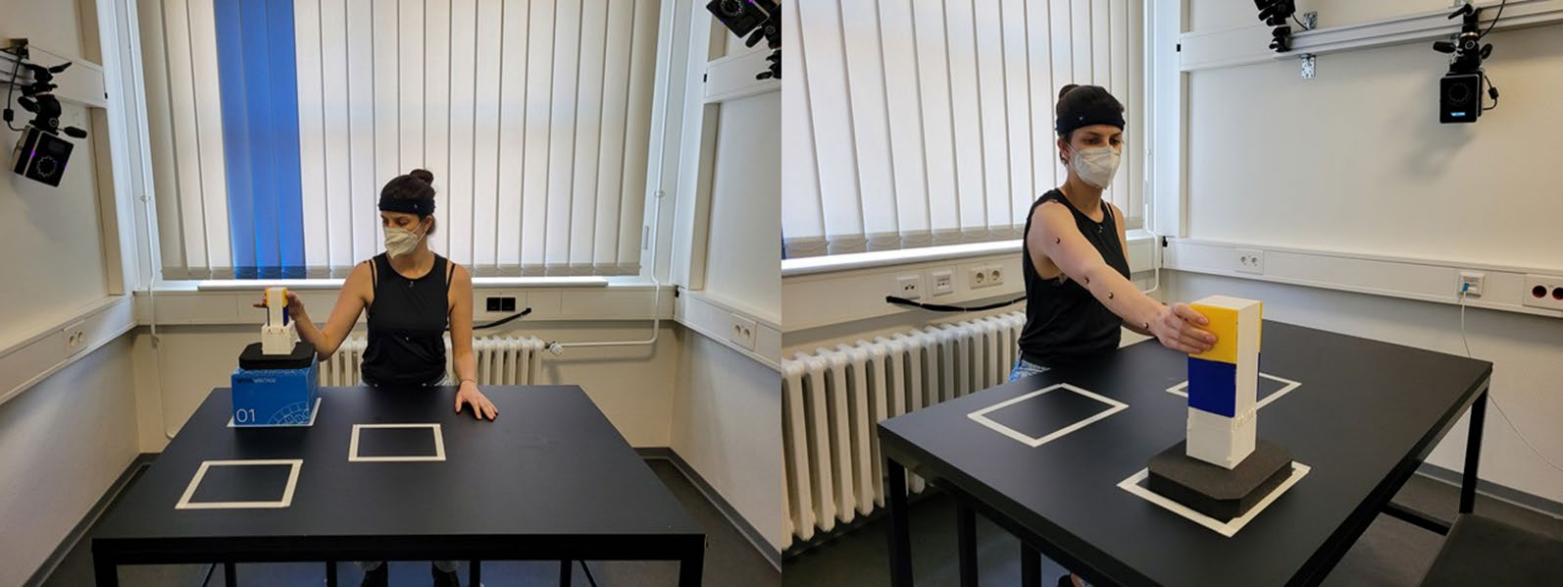
Experimental setup with both object sizes. Left: small object, near, elevated. Right: large object, far, on the table. The object was placed in one of the starting positions marked by the white rectangles on the right-hand side. The rectangle immediately in front of the participant marked the end position of the object

**Fig. 4:**
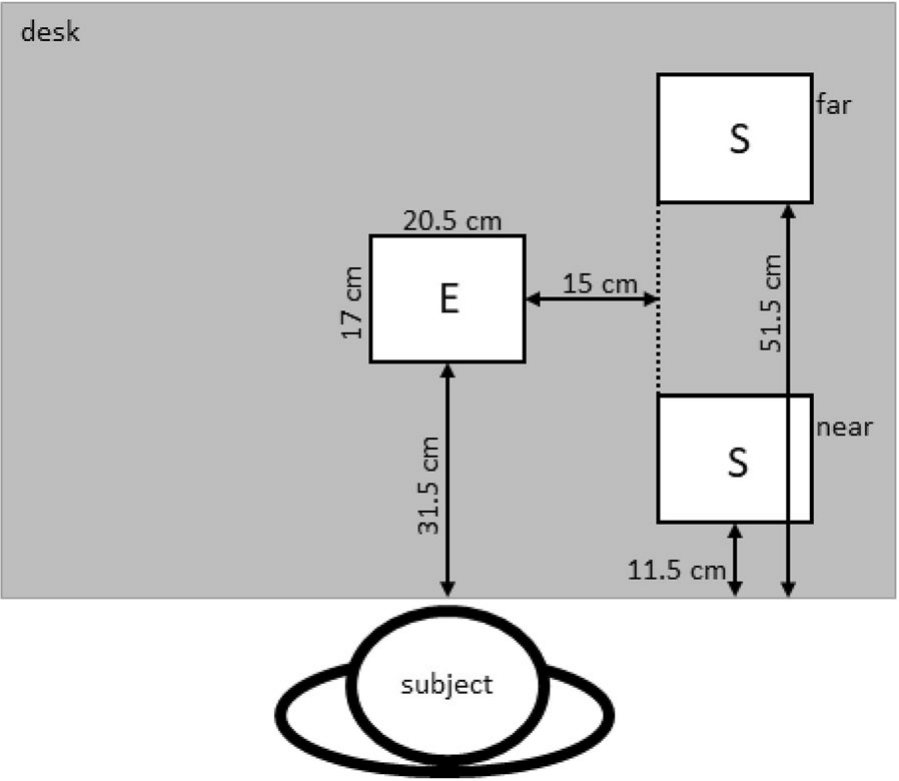
Task setup. Position of the start (S) and end (E) locations

Four different object starting positions (near/elevated, near/on the table, far/elevated, far/on the table) were used to ensure some variance in the arm movements. The start position of the object was varied in height (3 cm or 18 cm above the table surface, see Fig. 3) and distance (see Fig. 4). The target position where the object was to be placed remained fixed, a ${20.5}\,\hbox {cm} \times {17}\,\hbox {cm}$ area marked with tape and centered in front of the participant (see Fig. 3). Four blocks of 20 trials were performed ($M = 80$), so that each set of conditions was repeated 10 times (4 start positions $\times$ 2 weight classes) in pseudo-randomized order across the trials. The Vicon cameras were recalibrated before each block, which required the participant to stand up and leave the recording field. Accordingly, participants had approximately 5 min during each inter-block interval in which they could stand and move freely around the room.

At the start of a trial, the participant sat in the start position, the Vicon measurement was initiated and the object was on a foam pad placed in the object start position. The object was then switched on (so that the infrared LEDs could be tracked) and the experimenter said “okay” signaling to the participant that they could start the pick-up and replacement task. The participant then moved their right hand to the object, grasped it by the upper grasping surface and placed the object in the target position on the table. Participants were free to choose the grasp type and the number of fingers with which they grasped the object, with the only restriction being that they only touched the yellow surfaces of the object. The participant then moved back to the start/end position and the experimenter stopped the Vicon measurement. In total, each session lasted about 2 h.

### Analyses

#### Data preprocessing

The recorded data consisted of time series for the object velocity or the measured upper body angles, i.e.,  $\alpha _i$ for $i=1,\ldots , N$. For studying the velocity profiles of the object, we calculated the velocity of a point in the middle of the object, to be able to neglect the rotation of the object in z-direction. In order to compare the time series, they were each cut to their start and end point. The start point was taken as the first time stamp in which increase in the object’s height was detected. We then tested ending the time series with two different approaches: The first approach was to study the time series at the beginning of the lifting process and to use the first *T* measured points. Hence, we modeled each time series using the functions

1 $$\begin{aligned} \alpha _{i}(t), \quad t\in [0,1], \quad i=1,\ldots N, \end{aligned}$$

which we sampled at the discrete time points

$$\begin{aligned} { 0,\tfrac{t_2-t_1}{t_T-t_1},\tfrac{t_3-t_1}{t_T-t_1},\ldots , \tfrac{t_{T-1}-t_1}{t_T-t_1},1.} \end{aligned}$$

For comparison, we used a second approach where we cut the time series to the time $t_e$ when the objects’ height reached its final position as determined by the measurement of the objects’ height. This resulted in time series of different lengths for each trial. The mean length was 163 time stamps, i.e., 1630 ms, the minimum and maximum trial lengths were 1090 ms and 2260 ms, respectively. The different trial lengths were normalized to 1, where we used for every trial instead of the given time stamps $t_1,\dots , t_e$ the normalized time stamps.

**Fig. 5:**
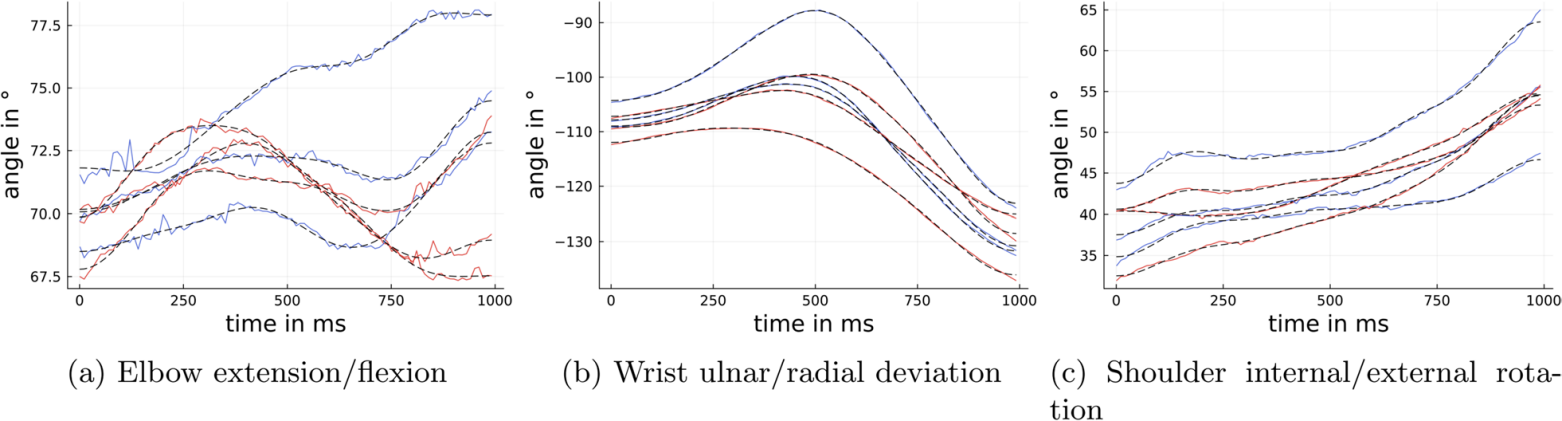
Exemplary time series data. Solid: data $\alpha _i(t)$ of one person for three of the seven angles. Black, dashed: Approximated time series ${\tilde{\alpha }}_i(t)$ with $n=8$. The colors belong to the heavy (red) and light (blue) object. We show the case $T=100$, i.e., using the first second of the time series. For every angle we show three trials for the heavy and the light object, respectively

Any missing data were interpolated linearly. An example based on the upper body angle time series, $\alpha _i(t)$, of one participant’s trials, using the first $T=100$ data points is plotted in Fig. 5.

#### Prediction procedure

The aim was to extract the information from the time series, $\alpha _i(t)$, which classifies the object’s weight. We either used the three velocity directions of the object, which means *N* = 3 or all the angles of the right arm, which gave a total of *N* = 7 angles. There were several reasons why it was not feasible to use the raw measured time series for classification. First and foremost, the measurements were noisy and had to be smoothed. Using all, approximately $163\cdot 3 = 489$ or $163\cdot 7 = 1141$, data points for each trial would have provided far too many features given information was collected for 960 trials. Furthermore, at specific times, the angles were not robust against changes in absolute position and the same movement from a slightly different starting position could result in completely different angles.

Therefore, we performed the following feature extraction: each time series, $\alpha _i(t)$, can be viewed as a smooth function defined on [0, 1], which facilitates the decomposition in basis functions. The half-period cosine basis is particularly useful in this case as it allows for every angle, *i*, to approximate the corresponding time series well by

2 $$\begin{aligned} \alpha _i(t) \approx \sum _{k=0}^{n-1} a_k^{(i)} \cos (\pi k t) =: {\tilde{\alpha }}_i(t), \quad i=1,\ldots {N}. \end{aligned}$$

The half-period cosine basis is a good choice for the approximation of non-periodic functions, as in this case, the decay rate is ${\mathcal {O}}(n^{3/2})$ and the coefficients $a_k^{(i)}$ can be calculated easily and quickly at specific points in each time series using the discrete cosine transform [25, Chapter 6]. Therefore, we described the time series $\alpha _i(t)$ using $n=8$ parameters $a_k^{(i)}$, $k=0,\ldots ,n-1$ which is, in contrast to the full time series, a very compressed expression. Another advantage of this procedure is that the sum of cosine functions smooths out any inaccuracies in the measurements that caused noisy data in the original time series. In Fig. 5, we used dashed lines to plot the approximate times series ${\tilde{\alpha }}_i(t)$ for $n=8$ in addition to the measured time series $\alpha _i(t)$ for the upper body angles approach. As seen in Fig. 5, the 8 parameters per time series appropriately captured the behavior of the functions, while fewer parameters would cause bigger discrepancies between the measured and approximated times series. For that reason, we worked with $n=8$ in the following analysis. Similar choices of *n* did not affect the results significantly.

Overall, for $M=80\cdot 12 = 960$ trials, we had $n=8$ parameters for each of the N time series. This can be considered as data compression. In other words, we received a matrix $\textbf{X}\in {\mathbb {R}}^{{(nN)} \times M}$ containing the parameters $a_{k}^{(i)}$ for every trial. Furthermore, we had the label vector $\textbf{y}\in \{-1,1\}^M$, which assigns $-1$ to a trial with low weight and 1 to a trial with high weight.

For the classification, we used Julia’s SVM, which is contained in LIBSVM [26] in the machine learning package. Different strategies for a train/test split and CV were used to evaluate how good our classification was. First, when performing the CV for *all trials*, we split all trials randomly 80/20 in training and test data sets. In a second analysis (CV *person-wise*) we used the trials from 2 participants as the test set and the trials from all other participants as the training set. In general, we standardized the values in $\textbf{X}$ in the training trials using a Z-transformation that transformed the mean and the variance of every column of $\textbf{X}$ to zero and one, respectively. It was necessary to do this because the parameters $a_{k}^{(i)}$ were scaled differently. In the prediction of the trials in the test set we had to transform the values in $\textbf{X}$ belonging to the test trials by the same transformation like the training set.

## Results

**Fig. 6:**
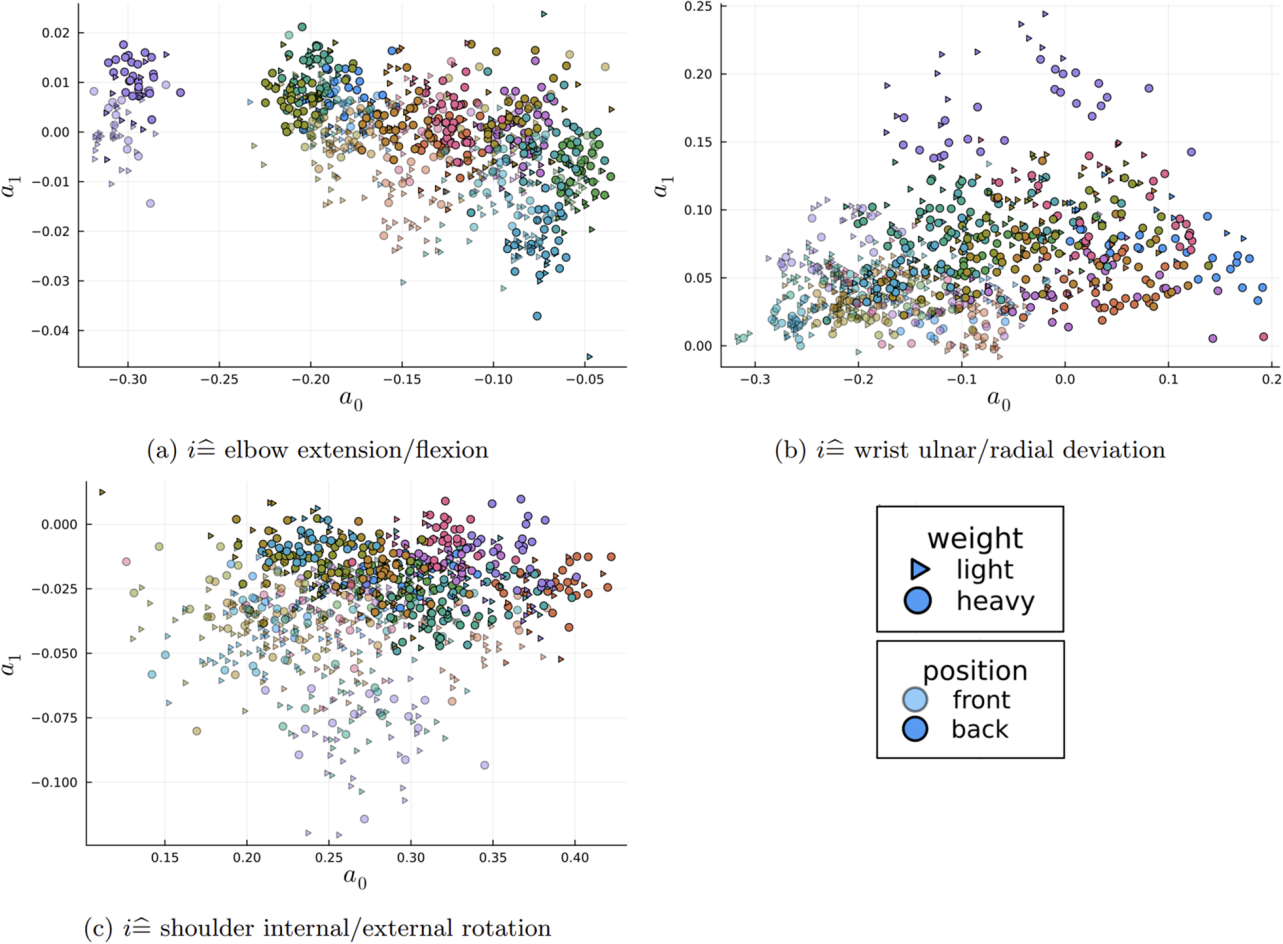
Parameter distribution. The parameters $a_{k}^{(i)}$ for $k=0,1$ for three of the seven different angles and all $M=960$ trials. The different colors belong to trials from different persons. The shape belongs to the object’s weight. The two different transparencies belong to the different starting positions front/back. We show the case T=100, i.e., using the first second of the time series

The parameters $a_k^{(i)}$ cluster by person as shown in Fig. 6. It can also be observed (especially in Fig. 6a) that the parameters cluster with respect to the object’s start position (near vs. far). Thus, the individual and the starting position both significantly affect the parameters. Hence, in order to predict the object weight class, which is depicted in Fig. 6 by the shape of the markers, it is necessary to identify differences in the parameters $a_k^{(i)}$ for the two different weights. Therefore, as described in Sect. 2.3, an SVM as a machine learning approach was used for classification.

### Object weight prediction using upper body angles

**Fig. 7:**
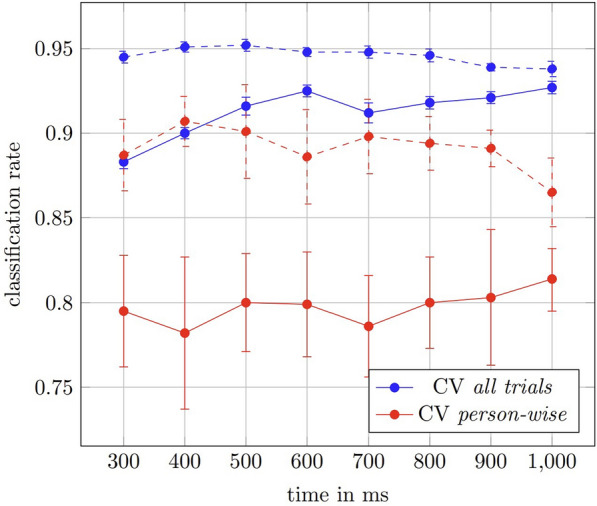
Mean classification rates. Mean classification rates of object weight prediction out of upper body angles (solid lines) versus out of velocity profiles of the object (dashed lines) using different CV strategies and different length of the grasp-process. The solid lines belong to the prediction from the upper body angles of the participant. For comparison, the dashed lines belong to the prediction from the velocity profiles of the object. The vertical lines depict the standard deviation of the classification rates, respectively

Using the prediction strategy described in Sect. 2.3, we attempted to determine whether the object was light or heavy based on the participant’s upper body angles. The results of the classification rates, as determined by the CV for various times (*T*) in the time series, are summarized in Fig. 7 (solid lines). We always conducted the CV procedure 10 times. The mean classification rate is shown Fig. 7.

As shown in Fig. 7, the results of the SVM returned a successful prediction rate of over $90\,\%$. This supports our hypothesis that the upper body angles, especially the parameters of the cosine transform include information about the object weight class. In relation to the best time point to predict the object weight, we revealed that, overall, the prediction accuracy was good ($88.3\%$ *all trials* or $79.5\%$ *person-wise*) even within the first 300 ms. The highest prediction accuracy ($92.7\%$ *all trials* or $81.4\%$ *person-wise*) was achieved within the first second. The prediction accuracy after 1 s was more reliable than the prediction made based on the complete time series ($87.3\%$ *all trials* or $78.5\%$ *person-wise*).

Two different CV procedures were performed: the *all trials* CV procedure led to a higher prediction accuracy than the *person-wise* CV procedure at all points in the time series.

### Object weight prediction using object velocity

**Fig. 8:**
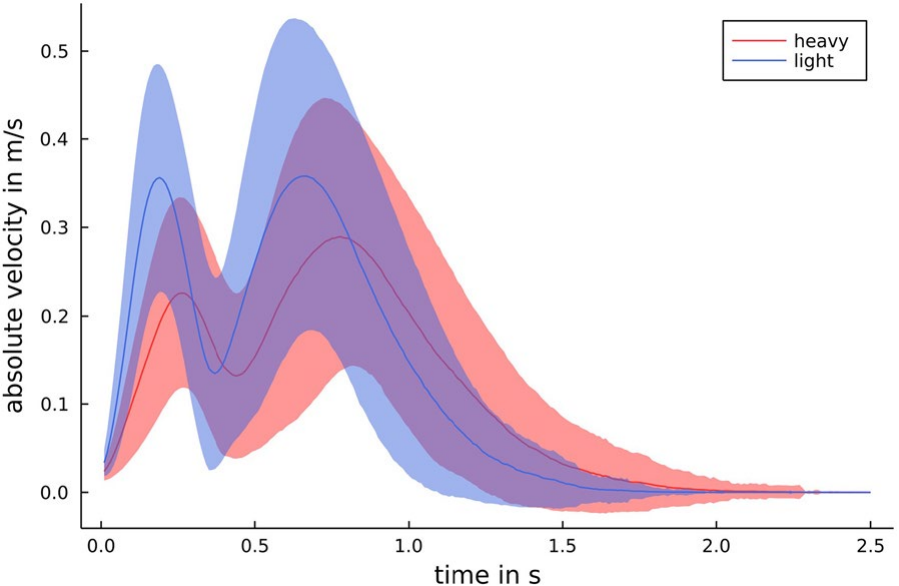
Object velocities. The mean of the absolute values of the object velocity in *z*-direction for all trials for the different weights of the objects. The standard deviation is shaded, respectively

**Fig. 9:**
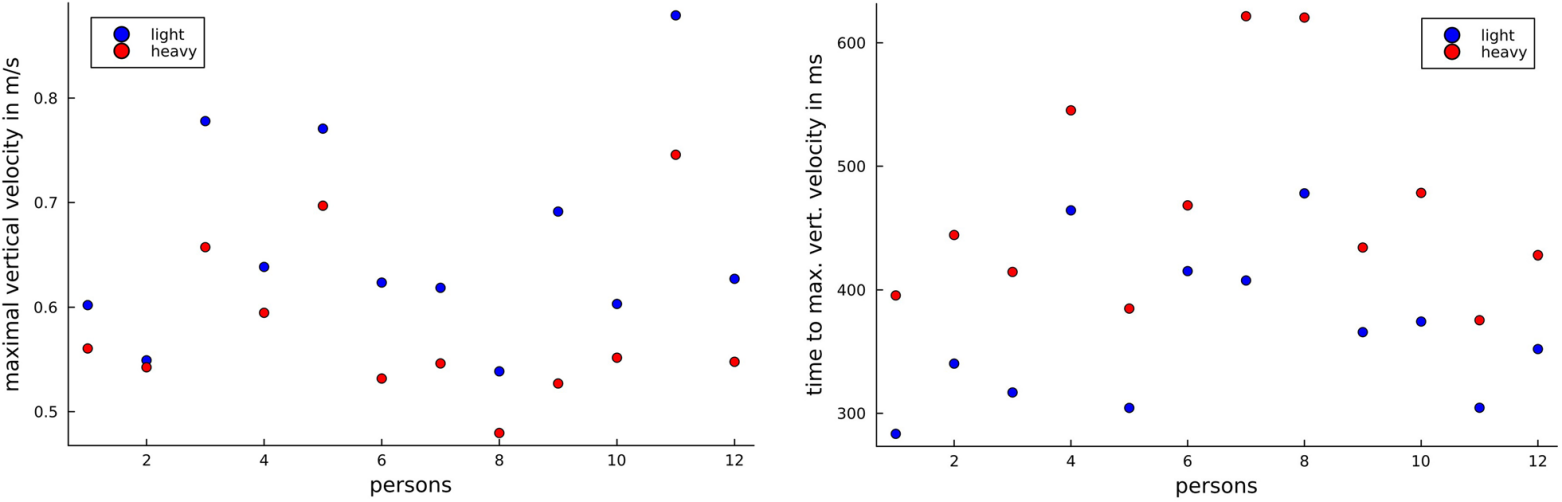
Maximal vertical velocity The mean of the maximum vertical velocity of the object in *z*-direction (left) and the mean time for reaching this maximum (right) for the two object weight classes (person-wise)

It was expected that the absolute lifting velocity of the object would change with the random change of the object weight class. Our results confirmed this (Fig. 8). We observed that light objects are lifted faster, which means that there is a difference in the velocity for lighter and heavier objects at the beginning of the lifting process. This was observed in all participants. For comparison, we give in Fig. 9 the mean maximum vertical velocity for every person with respect to the object weight class. These maxima differ for all persons, but there is no universal threshold for all persons, which allows a classification with the same accuracy as our approach. A universal threshold gives prediction accuracy of about $70\%$. So, the prediction accuracy benefits from the machine learning approach.

The results of this prediction from object velocities are also shown in Fig. 7 (dashed lines). Considering the CV results at the different points in the time series, together with the results of both CV procedures (*all trials* and *person-wise*), the total performance of the prediction based on object velocity was better than that based on upper body angles. In the *all trials* CV procedure, the object-based prediction reaches an accuracy of more than $95\%$ and is also nearly $95\%$ for all time series lengths *T*. Also in the *person-wise* CV test, the object-based prediction led to better performance of more than $90\%$. Also the standard deviations for the object-based predictions is smaller compared to upper body angle-based approach. In contrast: The prediction made based on the normalized complete time series led to similar prediction accuracy (86.4 % all trials or 78.5 % person-wise) as the prediction using upper body angles.

## Discussion

In this study, we investigated whether the weight class of an object can be predicted based on upper body and object kinematics during an object replacement task and whether the prediction can be successful regardless of the participant and the object’s starting position. Therefore, we used discrete cosine transformation for smoothing and data compression as well as SVM for classification using basis function parameters. Furthermore, we investigated how different (i) data lengths and (ii) CV procedures affect prediction accuracy.

The results showed that varying the object’s start position and the individual characteristics of participants have a large influence on the upper body profile of each move. Nevertheless, the object weight class was predicted with a high classification accuracy rate based on the upper body angles or object velocity, both when considering ${300}\,\hbox {ms}$ after the participant’s first contact with the object and the entire object transport in a time-normalized manner.

### Classification accuracy in other studies

To predict the object weight class, we used upper body angle time series. We could not find any study in which object properties were predicted based on kinematics in a pick-up and replacement task. However, previous studies have investigated machine-learning approaches and classification based on human movement kinematics in other domains. For example, Shetty and Rao [13], proposed an approach to detect Parkinson’s disease based on gait kinematics and achieved an accuracy of about 83%. Classification procedure in Shetty and Rao [13] differed remarkably from our approach. They selected individual temporal parameters within the gait data, such as the duration for which both feet are in contact with the ground in a gait cycle. In addition, relative gait data were taken into account, such as the percentage of time in a gait cycle when both feet are in contact with the ground. When comparing the prediction accuracy, which was similar to that of our approach, it should also be noted that the participants in Shetty and Rao’s [13] study were known Parkinson’s patients while the participants in our study were healthy young adults. Considering that the gait quality of a Parkinson’s patient is usually very different from that of healthy subjects, it can be assumed that the discrimination should not be a major challenge for a machine-learning approach. Accordingly, we consider the classification accuracy we obtained from a homogeneous group as stronger than the classification accuracy achieved by Shetty and Rao [13]. Furthermore, it should be kept in mind that we were able to show, as other studies have [22, 27–30], that the kinematics of each individual are distinct. As a person is assigned to either the patient or healthy group and cannot switch between these roles, individual factors (unrelated to Parkinson’s disease) may improve classification. However, this would only increase the performance in a sample study, not in the field.

The only study, we are aware of that has also examined arm kinematics was undertaken by Bosco and colleagues [31]. Participants in that study were asked to perform grasping movements towards visually indicated targets. Bosco and colleagues [31] were able to predict performance (trajectory deviation) from the kinematics of grasping movements. For this purpose, both healthy participants and a participant with a parieto-occipital lesion were tested. Based on the performance, the researchers were able to distinguish the patient from the healthy subjects using a linear discriminant function. Overall, the prediction accuracy varied between 75% and 95% and a prediction accuracy of about 90% was achieves after only 20% of the movement had been executed. They analyzed arm movements using a much simpler classification process. Yet, the prediction accuracy for the distinction between patients and healthy subjects was very high. However, as in the study by Shetty and Rao [13], it can be assumed that this distinction was not as demanding as it would have been in a homogeneous group. The prediction accuracy of the healthy subjects was around 80%. Therefore, our results show higher accuracy in comparison to this study as well.

### Accurate classification can be achieved as early as 300 ms

One of our aims was to evaluate the classification accuracy depending on the length of the time series (e.g., whole time series vs. the first 300 ms of the time series). Our results show that this approach achieved a good prediction accuracy within the first 300 ms, with the highest prediction accuracy achieved when considering the first second. This result is in line with previous studies that have shown that the early part of the lifting phase is particularly valuable for object’s weight prediction [3, 32]. That is, the prediction was more reliable based on the data from the first second of the movement than if the entire trial was taken into account. We suggest that this is due to the fact that time-normalization was necessary to compare time series of different lengths. Within this normalization some information about time and velocity was lost. In contrast, when using the first *T* time series data points, velocity information was retained. Furthermore, we assumed that considering the entire movement included more noise, which led to a lower prediction accuracy.

One likely explanation is that important information needed for the prediction of the object’s weight class is contained in the beginning of the lifting movement. When a receiver observes a giver lifting an object prior to a joint handover action, adjustment of the internal model is possible as early as 300 ms into the movement. That is, the prediction accuracy improves within the following 300 ms (see Fig. 7), after which it deteriorates. This means that robots should focus, primarily, on the first 600 ms in total instead of seeking to observe the whole action. This result also suggests that robots (intended to perform joint handover actions with humans) should be designed with a focus on observing the start of a lifting movement when making weight class predictions and calculating the necessary grip and load forces. In addition, this would have the additional advantage that, after the initial prediction there is still time to carry out the calculation of the grip and load forces.

Being able to predict object weight class at an early point in the action makes this approach a promising possibility in human–robot interaction. For example, if a robot is able to classify the object properties at such an early point in the handover action, this may facilitate an online adaptation of the required execution.

### Person-wise CV yields poorer classification accuracy than all trials CV

When analyzing Fig. 6, we noticed that the data seems to be clustered by person, which is presumably due to participant anthropometry on the one hand, but also indicates a high degree of individuality in the movement. This individuality occurs even though the data were recorded under controlled laboratory conditions. This observation has also been made in previous studies in other actions, including gait [27–30], eye movement [33], and facial movement [34]. Moreover, this individuality of movement has also been detected in hand movements or, more precisely, handover actions [22]. We assume that this individuality is also the reason for the variation in prediction accuracy between CV procedures. If the training and test data were split between persons (*person-wise* CV), this led to a generally lower prediction accuracy compared to randomly splitting the data across all participants (*all trials* CV). Due to the inter-individual differences in movements, the prediction works well when the data from one person are contained in both the training and test data. However, in order to implement this prediction approach in possible joint actions between humans and robots, we need to obtain classification strategies that are as universally valid as possible. This would make it possible to run spontaneous actions with different people smoothly without the need for a prior learning phase. In order to avoid this application issue, particularly large training data sets, based on as many different people as possible, should be used. Therefore, we suggest that this approach be tested in more complex setups and with a variety of different people to test whether our results can be generalized.

### The highest classification accuracy achieved by observing object velocity profiles

Our study revealed, that the weight class of an object can be classified by observing the upper body angle profiles of a person grasping and replacing the object or observing the object velocity profiles. Overall, our classification accuracy based on object velocity profiles was higher than for our classifications based on upper body angles. This could be because the object weight class was changed randomly, i.e., the participants did not know the weight class of the object in any given trial. Therefore, they could not anticipate the lift force required, which resulting in greater difference in lifting velocities depending on the object’s weight class [8, 9]. This indicates that, there is a large amount of information contained in object velocity , which is consistent with earlier findings [3, 32, 35]. This conclusion is supported by Figs. 8 and 9 which shows that the velocity profile, especially the vertical dimension, in a replacement task are influenced by the object weight class. Furthermore, this result is consistent with a previous study by Johansson and Westling [8] which demonstrated that the maximum lifting velocity depends on object weight. The prediction accuracy of the object velocity-based approach might be reduced if the person is aware of the object’s weight class and can anticipate the load force required. It follows that further studies should examine whether the proposed object weight class prediction approach is robust to the person’s awareness of the object’s weight class. Furthermore, it is demonstrated (see Fig. 7) that the CV procedure has less influence on the object velocity-based classification than on the upper body angle-based approach. We assume that the CV procedure has a minor influence on the prediction accuracy of the object-based approach, as interpersonal physiological differences are negligible when considering object velocity alone.

### Limitations

It should be noted that in this experiment a very simple and standardized action was performed, namely a pick-up and replacement task. However, everyday life involves much more complex object transport actions (e.g., handover actions). In contrast to the experiment conducted here, more time-critical actions and variations in the object’s end position increase the variance in a person’s movement. Therefore, we propose that this approach should be tested further in relation to more complex actions.

Furthermore, it should be noted that the upper limb Plug-in Gait model is a simplified model that is proposed for the study of arm movements during gait, but it is unclear how well the calculated angles are suited for a comprehensive consideration of arm kinematics. Nevertheless, the results of our study show that, even using this simplified model, predictions about the object weight class can be made with high accuracy.

Only two different weights were used in this study, i.e., only a distinction was made between the two object classes “light” and “heavy”. Therefore, it is difficult to assess whether it is possible to estimate the weight of an object based on the arm kinematics. To overcome this limitation, we suggest that future studies should investigate whether the approach presented here also works with a higher number of different weight classes and finer gradations between the weight classes. Such further research could then indicate whether and, if so, how the estimation of object weight based on upper body angles could be implemented in such actions.

Due to the random variation in object weight class, the participants could not develop an accurate internal model for the object property weight. Accordingly, it can be assumed that a flawed forward model was used to generate grip and load forces each time the task was performed. This would have necessitated a large feedback correction. This feedback correction is reflected in the results of the object velocity in z-direction (Fig. 8) and we observed the expected effect of excessive lift velocity and height during the trials involving the lighter object weights. It is possible that the velocity profile of the lifting movement was influenced more by the conditions (light vs. heavy) than would have been the case if the participants had known the object weight class before performing the task. Therefore, we suggest that future studies include an additional control block in which the participants have to replace the light and then heavy object in a blocked condition.

## Conclusion and future directions

In this study, our aim was to predict the object weight class using time series of the upper body angles and object velocities in a replacement task. We found that it was possible to distinguish between “heavy” and “light” objects with a high prediction accuracy, even very early (after 300ms) in the execution of the movement. Furthermore, it was shown that the accuracy of the prediction depends on the CV procedure used, that is, splitting the data across *all trials* leads to a higher accuracy than splitting by person due to the significant inter-individual differences. This raises the question of whether the prediction approach can become reliable enough for robot–human interactions, or whether more emphasis should be placed on individualized analysis. The prediction based on object velocities was less affected by the CV procedure and provided higher overall accuracy.

This approach enriches robotics research in the field of human–robot interaction. In order to make actions between humans and robots (e.g., handover actions) as intuitive as possible, robots need to be able to estimate the weight of an unknown object in order to anticipate the grip forces needed in any given situation. To improve this ability, the approach presented here could supplement and improve the adaptability of current methods such as image recognition [7] and thermography [4].

## Data Availability

The datasets used and analyzed during the current study are available from the corresponding author on reasonable request.
